# Pulsating electromagnetic fields for perineal lacerations and surgical wounds healing in the postpartum: a pilot study

**DOI:** 10.1007/s00404-024-07671-3

**Published:** 2024-08-21

**Authors:** Ilma Floriana Carbone, Francesca Maria Paola Gigli, Gabriele Rossi, Valentina Romagnoli, Benedetta Gallicola, Flavia Sandi, Giovanna Esposito, Enrico Mario Ferrazzi

**Affiliations:** 1 Unit of Obstetrics, Department of Woman, Child and Neonate, Fondazione IRCCS Ca’ Granda Ospedale Maggiore Policlinico, Mangiagalli Center, Milan, Italy; 2 Department of Clinical and Community Science, Dipartimento di Eccellenza 2023-2027, University of Milan, Milan, Italy

**Keywords:** Therapeutic Magnetic Resonance, Perineal tears, Episiotomy

## Abstract

**Purpose:**

The aim of our study was to assess the possible benefits of Therapeutic Magnetic Resonance (TMR) in the treatment of spontaneous perineal lacerations and episiotomies in the postpartum.

**Methods:**

We performed a prospective, non-pharmacologic, non-profit, monocentric interventional study on women who had a spontaneous laceration and/or an episiotomy at delivery. The TMR device treatment was accepted by 52 women, while 120 women underwent standard care. Patients were visited 1 day postpartum, before starting the treatment; then a follow-up visit was performed at 3 weeks, 5 weeks, and 3 months after delivery. The main endpoint was the time required for complete healing of the laceration and/or the episiotomy. Secondary endpoints were the prevalence of dehiscence, infections, urinary discomfort, urinary leakage, and the quality of restoration of sexual function.

**Results:**

In the treatment group the REEDA score was significantly better both at 3- and 5-weeks postpartum follow-up. At 3 weeks and 5 weeks postpartum, we observed a significantly better outcome in the treatment group for all subjective complaints and perineal complications associated with lacerations and episiotomies. The percentage of patients who scored above the cutoff for sexual dysfunction was significantly better in the treatment group (83.3%) than in the control group (31.8%) (p < 0.001).

**Conclusions:**

With this pilot study, we introduced low dose Pulsating Electromagnetic Fields (PEMFs) as a novel conservative and not pharmacological approach to reduce complications of perineal lesions. Our results demonstrated to significantly improve perineal wound healing and to ameliorate the sexual function in the postpartum.

## What does this study add to the clinical work

**Table:** 

We introduced low dose Pulsating Electromagnetic Fields (PEMFs) as a novel conservative and not pharmacological approach to reduce complications of perineal lesions.	

## Background

In the last years, in the context of surgical healing techniques, there has been a growing interest in non-invasive biophysical treatments to support pharmacological therapies. Among these, the Pulsating Electromagnetic Fields (PEMFs), and in particular the Therapeutic Magnetic Resonance (TMR), which had originally been studied in orthopedics by Basset et al. [1], look promising. After the approval by the FDA in 1979, this technology has been investigated in other medical domains, including traumatology [2–8], neurology [9, 10], oncology [11–15] and superficial wound healing. Possible applications in this latter field include the healing and microvascular circulation of chronic diabetic foot ulcers [16–18] and reducing postoperative pain after breast plastic surgery [19].

TMR is based on the exposition of PEMFs, which work at molecular, cellular, and tissue level. At molecular level, there are effects on ionic particles, molecules, macromolecules, intracellular concentration of calcium ions and free radicals [9, 20–23], epigenetic modifications of the transcription process of DNA [24–26], and at mitochondrial level, effects on the electron transport chain [27]. At cellular level, there are effects on microcrystals, membranes, and cellular organelles, as the upregulation of ion channels [28], the modulation of apoptosis [29–31], and the expression of adenosine receptors A2A and A3 [32–34]. At tissue level, these effects achieve a reduction of inflammatory processes [19, 35–37] and an improvement of microcirculations [38].

Spontaneous perineal lacerations and episiotomies are common during childbirth. Perineal lacerations occur in 53–79% of vaginal deliveries [39, 40] , while episiotomies vary significantly from one country to another, ranging among European countries between 3,7% in Denmark to 75% in Cyprus [41].

These lesions might be associated with short and long-term physical complications such as bleeding, pain, hematomas, infections, suture dehiscence, and dyspareunia, that can also lead to negative psychological effects [42–46].

We hypothesized that the positive results of low intensity PEMFs could be applied after vaginal birth. The aim of our study was to assess the possible benefits of this technology in the treatment of spontaneous perineal lacerations and episiotomies in the postpartum.

## Methods

### Study design, setting and population

We performed a prospective, non-pharmacologic, non-profit, monocentric interventional study. This pilot study was conducted on women who delivered and were admitted to the postpartum ward of Mangiagalli Center High risk Obstetric Unit—Ospedale Maggiore Policlinico of Milan between 8th January to 8th August 2023.

Each consecutive Wednesday, we recruited patients who suffered either a spontaneous laceration and/or an episiotomy at delivery. Exclusion criteria were presence of clinical signs and /or symptoms of infection of the suture, pregestational or gestational diabetes under pharmacologic therapy, autoimmune diseases, other diseases that could interfere with the process of wound healing.

Fort this study  172 women were eligible, but only  52 women accepted to participate to this pilot study with TMR device (registered trademark Diapason by Thereson®) (treatment group). The treatment was declined by 120 women, but they accepted standard care (control group) and follow-up according to the same schedule as those treated by TMR.

Treatment with TMR device required two sessions per day for 15 days. During the treatment, patients were lying down on the body emitter mat, while the local, smaller therapy emitter mat was positioned on the lower abdomen to cover the pubic bones and pelvis. Each treatment consisted of two phases, for a total of 32 min. During the first phase (“Local phase”), which lasted 16 min, the intensity ranged between 150 and 250 µT, and the electromagnetic fields were focused on the pelvic area. During the second phase (“Total phase”), which also lasted 16 min, the intensity ranged between 130–250 µT and the electromagnetic fields were focused at different locations (chest, abdomen, and lower limbs). Upon discharge form the Hospital, patients were provided with a TMR device to perform the treatment at home. Standard care was based on perineal hygiene with the addition of a topic spray until complete healing of the suture.

Patients were visited one day postpartum, before starting the treatment; then a follow-up visit was performed at 3 weeks (time 1), 5 weeks (time 2), and 3 months (time 3) after delivery.

To determine the status of the wound before the treatment, one day postpartum, and to assess the progression of healing at the 3- and 5-weeks follow-ups, we assigned to each patient a score, based on the REEDA (Redness, Edema, Ecchymosis, Discharge, Approximation) scale [47] (Table 1), which can be used to assess all types of postpartum perineal trauma. For each assessed item, a score ranging from 0 to 3 can be assigned by the healthcare provider. A higher score indicates a greater level of tissue trauma.

**Table 1:** The parameters of the REEDA (Redness, Edema, Ecchymosis, Discharge, Approximation) scale

	Redness	Edema	Ecchymosis	Discharge	Approximation
0	None	None	None	None	None
1	Within 0.25 cm of the incision bilaterally	Perineal, less than 1 cm of the incision	Within 0.25 cm bilaterally or 0.5 cm unilaterally	Serous	Skin separation 3 mm or less
2	Within 0.5 cm of the incision bilaterally	Perineal and/or 1–2 cm from the incision	Between 0.25 and 1 cm bilaterally or between 0.5 and 2 cm unilaterally	Sero-sanguineous	Skin and subcutaneous fat separation
3	Beyond 0.5 cm of the incision bilaterally	Perineal and/or vulvar, greater than 2 cm from the incision	Greater than 1 cm bilaterally or 2 cm unilaterally	Bloody, purulent	Skin, subcutaneous fat and fascial layer separation

At the 3- and 5-weeks follow-up visits, in addition to the healing progression, we also evaluated other possible complications and complaints, such as urinary discomfort and urinary leakage, infections, suture dehiscence, and fistulas.

At 3 months after delivery, patients were contacted via e-mail to answer the FSFI (Female Sexual Function Index) questionnaire [48, 49] (Table 2). This questionnaire collects self-reported answers on six sexual domains (desire, arousal, lubrication, orgasm, satisfaction, and pain) and can provide a key to interpreting Female Sexual Dysfunctions. The total score is a sum of each domain score, ranging from 2 to 36 (Table 3), where a score below 26.5 is a good indicator of sexual dysfunction [50].

**Table 2:** The Female Sexual Function Index (FSFI) questionnaire

Questions	Answers
1. Over the past 4 weeks, how often did you feel sexual desire or interest?	a. Almost always or always b. Most times (more than half the time) c. Sometimes (about half the time) d. A few times (less than half the time) e. Almost never or never
2. Over the past 4 weeks, how would you rate your level (degree) of sexual desire or interest?	a. Very high b. High c. Moderate d. Low e. Very low or none at all
3. Over the past 4 weeks, how often did you feel sexually aroused (“turned on”) during sexual activity or intercourse?	a. No sexual activity b. Almost always or always c. Most times (more than half the time) d. Sometimes (about half the time e. A few times (less than half the time) f. Almost never or never
4. Over the past 4 weeks, how would you rate your level of sexual arousal (“turn on”) during sexual activity or intercourse?	a. No sexual activity b. Very high c. High d. Moderate e. Low f. Very low or none at all
5. Over the past 4 weeks, how confident were you about becoming sexually aroused during sexual activity or intercourse?	a. No sexual activity b. Very high confidence c. High confidence d. Moderate confidence e. Low confidence f. Very low or no confidence
6. Over the past 4 weeks, how often have you been satisfied with your arousal (excitement) during sexual activity or intercourse?	a. No sexual activity b. Almost always or always c. Most times (more than half the time) d. Sometimes (about half the time e. A few times (less than half the time) f. Almost never or never
7. Over the past 4 weeks, how often did you become lubricated (“wet”) during sexual activity or intercourse?	a. No sexual activity b. Almost always or always c. Most times (more than half the time) d. Sometimes (about half the time e. A few times (less than half the time) f. Almost never or never
8. Over the past 4 weeks, how difficult was it to become lubricated (“wet”) during sexual activity or intercourse?	a. No sexual activity b. Extremely difficult or impossible c. Very difficult d. Difficult e. Slightly difficult f. Not difficult
9. Over the past 4 weeks, how often did you maintain your lubrication (“wetness”) until completion of sexual activity or intercourse?	a. No sexual activity b. Almost always or always c. Most times (more than half the time) d. Sometimes (about half the time e. A few times (less than half the time) f. Almost never or never
10. Over the past 4 weeks, how difficult was it to maintain your lubrication (“wetness”) until completion of sexual activity or intercourse?	a. No sexual activity b. Extremely difficult or impossible c. Very difficult d. Difficult e. Slightly difficult f. Not difficult
11. Over the past 4 weeks, when you had sexual stimulation or intercourse, how often did you reach orgasm (climax)?	a. No sexual activity b. Almost always or always c. Most times (more than half the time) d. Sometimes (about half the time e. A few times (less than half the time) f. Almost never or never
12. Over the past 4 weeks, when you had sexual stimulation or intercourse, how difficult was it for you to reach orgasm (climax)?	a. No sexual activity b. Extremely difficult or impossible c. Very difficult d. Difficult e. Slightly difficult f. Not difficult
13. Over the past 4 weeks, how satisfied were you with your ability to reach orgasm (climax) during sexual activity or intercourse?	a. No sexual activity b. Very satisfied c. Moderately satisfied d. About equally satisfied and dissatisfied e. Moderately dissatisfied f. Very dissatisfied
14. Over the past 4 weeks, how satisfied have you been with the amount of emotional closeness during sexual activity between you and your partner?	a. No sexual activity b. Very satisfied c. Moderately satisfied d. About equally satisfied and dissatisfied e. Moderately dissatisfied f. Very dissatisfied
15. Over the past 4 weeks, how satisfied have you been with your sexual relationship with your partner?	a. No sexual activity b. Very satisfied c. Moderately satisfied d. About equally satisfied and dissatisfied e. Moderately dissatisfied f. Very dissatisfied
16. Over the past 4 weeks, how satisfied have you been with your overall sexual life?	a. No sexual activity b. Very satisfied c. Moderately satisfied d. About equally satisfied and dissatisfied e. Moderately dissatisfied f. Very dissatisfied
17. Over the past 4 weeks, how often did you experience discomfort or pain during vaginal penetration?	a. Did not attempt intercourse b. Almost always or always c. Most times (more than half the time) d. Sometimes about half the time) e. A few times (less than half the time) f. Almost never or never
18. Over the past 4 weeks, how often did you experience discomfort or pain following vaginal penetration?	a. Did not attempt intercourse b. Almost always or always c. Most times (more than half the time) d. Sometimes about half the time) e. A few times (less than half the time) f. Almost never or never
19. Over the past 4 weeks, how would you rate your level (degree) of discomfort or pain during or following vaginal penetration?	a. Did not attempt intercourse b. Very high c. High d. Moderate e. Low f. Very low or none at all

**Table 3:** Interpretation of the FSFI score

Domain	Items	Score range	Factor	Min score	Max score
Desire	1, 2	1–5	0.6	1.2	6
Arousal	1, 3, 4, 5, 6,	0–5	0.3	0	6
Lubrication	7, 8, 9, 10	0–5	0.3	0	6
Orgasm	11, 12, 13	0–5	0.4	0	6
Satisfaction	14, 15, 16	1–5	0.4	0.8	6
Pain	17, 18, 19	0–5	0.4	0	6
Scale range				2	36

The main endpoint of the study was the time required for complete healing of the laceration and/or the episiotomy. Secondary endpoints were the prevalence of dehiscence, infections, urinary discomfort, urinary leakage, and the quality of restoration of sexual function.

### Statistical analysis

Descriptive statistics were used to summarize selected baseline characteristics of intervention and control group. Categorical data were presented as absolute frequencies and percentages, while quantitative data were expressed as mean and standard deviation. Differences in categorical variables between the two groups were tested by using Fisher test. Continuous variables were compared by using the t-test for independent samples.

The REEDA scores obtained from women in the treatment and control groups 1 day, 3 weeks, and 5 weeks postpartum were expressed as median and interquartile range (IQR), and differences were tested by Wilcoxon rank-sum test. The frequencies of selected adverse outcomes were reported separately according to treatment, and Fisher test was used to assess differences between the two groups.

Finally, results from the FSFI questionnaire were reported as both categorical (i.e. ≤ 26.5 and > 26.5) and continuous scores (expressed as median and IQR). Differences were tested with Fisher test and Wilcoxon rank-sum test.

## Results

The study included 172 patients with a mean maternal age of 33.5 years (range 18–46), the majority of whom were nulliparous (82.0%). They gave birth at an average gestational age of 39.5 weeks and the mean birth weight was 3320.0 g. Episiotomy was performed in 62.2% of cases and first, second and third degree lacerations occurred in 40.7%, 54.7% and 4.7% of cases respectively. 52 women underwent treatment by PEMFs with TMR device, while 120 women underwent standard care.

Table 4 shows demographic and clinical characteristics of the two groups. The women who underwent TMR were older, more likely to have had a vacuum-assisted birth using Kiwi-cup ®, and had more severe perineal lacerations. In the treatment group, 49 patients (94.2%) came to at least one of the 3- or 5-weeks follow-up, and 24 patients (46.2%) answered the FSFI questionnaire at 3 months postpartum. In the control group, 67 patients (55.8%) came to at least one visit at 3- or 5-weeks, while only 22 patients (18.3%) answered the FSFI questionnaire.

**Table 4:** Demographic and clinical data of the treatment group and the control group

Demographic and clinical data	Treatment group, N = 52	Control group, N = 120	p-value*
Maternal age	34.6 ± 3.3	33.0 ± 4.7	0.012
Nulliparous women	42 (80.8)	99 (82.5)	0.830
Spontaneous conceivement	49 (94.2)	111 (92.5)	> 0.999
BMI at term	26.2 ± 4.4	26.1 ± 3.4	0.818
Spontaneous labor	37 (71.2)	74 (61.7)	0.298
Gestational age at delivery (weeks)	39.3 ± 1.6	39.6 ± 1.8	0.248
Newborn weight at birth (g)	3325.1 ± 358.5	3317.8 ± 400.6	0.910
Total blood loss (mL)	422.1 ± 327.1	367.5 ± 249.0	0.285
Vacuum-assisted delivery (Kiwi-cup ®)	15 (28.8)	15 (12.5)	0.015
Episiotomy			
No	14 (26.9)	51 (42.5)	
Median episiotomy	1 (1.9)	1 (0.8)	
Mediolateral episiotomy	37 (71.2)	68 (56.7)	0.100
Spontaneous laceration			
No	30 (57.7)	56 (46.7)	
I degree perineal lacerations	4 (7.7)	31 (25.8)	
II-degree perineal lacerations	17 (32.7)	30 (25.0)	
III-degree perineal lacerations	1 (1.9)	3 (2.5)	0.035
Spontaneous laceration + episiotomy	7 (13.5)	13 (10.8)	0.613

Data are reported as mean ± standard deviation or absolute number (percentage), as appropriate

*Fisher test for categorical variables and t test for continuous variables

Table 5 shows the differences in wound healing, according to the REEDA score, between the treatment group and the control group. At baseline (1 day after delivery), no differences emerged (p = 0.874). The REEDA scores obtained at 3 (p = 0.005) and 5 (p < 0.001) weeks postpartum were lower in women who underwent TMR.

**Table 5:** Comparison between REEDA score in the treatment group and in the control group 1 day, 3 weeks, and 5 weeks postpartum

	REEDAscore		p-value*
Timing of follow-up	Treatment group	Control group	
One day after delivery	4 (3–5)	4 (3–4)	0.874
Three weeks after delivery	1 (1–3)	2 (2–3)	0.005
Five weeks after delivery	1 (0–1)	2 (1–2)	< 0.001

REEDA score is expressed as median (interquartile range, IQR)

*Wilcoxon rank-sum test

The complications of wound healing and other subjective complaints related to perineal dysfunctions at the 3- and 5-week follow-ups, are reported in Tables 6 and 7.

**Table 6:** Outcomes at 3 weeks postpartum

Adverse outcomes	Treatment group, N = 32	Control group, N = 61	p-value*
Non-complete healing	12 (37.5)	41 (67.2)	0.008
Dehiscence	1 (3.1)	15 (24.6)	0.009
Infection	0 (0.0)	1 (1.6)	> 0.999
Urinary discomfort	1 (3.1)	15 (24.6)	0.009
Urinary leakage	2 (6.3)	17 (27.9)	0.015

Data are reported as absolute number and percentage in brackets

*Wilcoxon rank-sum test

**Table 7:** Outcomes at 5 weeks postpartum

Adverse outcomes	Treatment group, N = 39	Control group, N = 42	p-value*
Non-complete healing	1 (2.6)	8 (19.1)	0.030
Dehiscence	0 (0.0)	0 (0.0)	NA
Infection	0 (0.0)	0 (0.0)	NA
Urinary discomfort	1 (2.6)	10 (23.8)	0.007
Urinary leakage	3 (7.7)	13 (31.0)	0.011

Data are reported as absolute number and percentage in brackets

*Wilcoxon rank-sum test

At 3 weeks postpartum, we observed a significantly better outcome in the treatment group in terms of healing, dehiscence, urinary discomfort, and urinary leakage. The only aspect that was not statistically significant was the occurrence of surgical site infections. Not any fistula occurred in this study. As for healing and urinary disorders, similar significantly better outcomes were observed at 5 weeks postpartum. At this time, no cases of dehiscence or infection were reported.

**Fig. 1:**
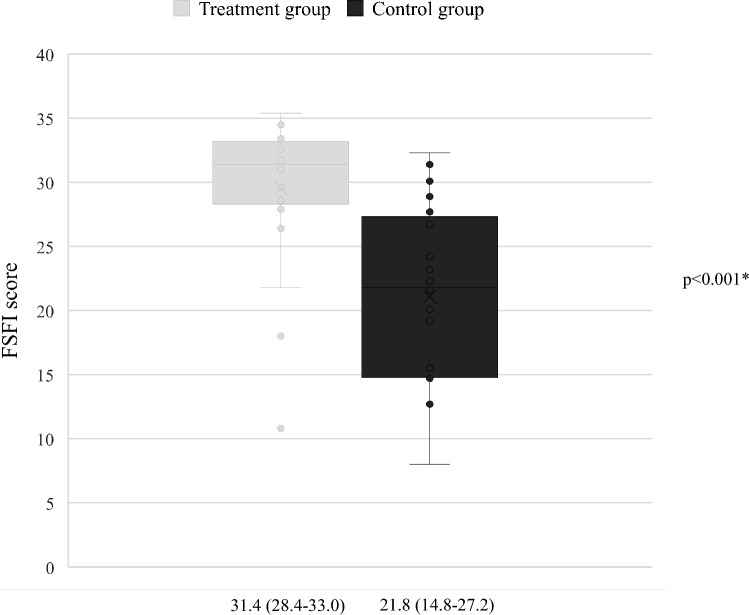
FSFI score is expressed as median (interquartile range, IQR). *Wilcoxon rank-sum test

Figure 1 shows box and whiskers plot of FSFI scores for the treatment group (light grey box) and the control group (dark grey box). The median FSFI score was 31.4 (28.4–33.0) in the treatment group and 21.8 (14.8–27.2) in the control group (p < 0.001). With the limitation of the cases lost to follow-up, the number of patients who scored above the cutoff for sexual dysfunction (i.e., 26.5) was significantly higher in the treatment group (20/24, 83.3%) than in the control group (7/22, 31.8%) (p < 0.001).

## Discussion

The main outcome of data we collected and compared in the intervention and in the control group confirmed that PEMFs achieved a significantly better progression of wound healing at 3- and 5-weeks postpartum according to the REEDA score. Similarly, complications of wound healing and other subjective complaints related to perineal dysfunctions at 5 weeks were significantly more reported in the control group despite the relative prevalence of first-degree perineal lacerations. The Female Sexual Dysfunctions proved a significantly better outcome in the treatment group.

With this study, we introduced for the first time in the clinical practice, PEMFs as a novel conservative and not pharmacological approach to reduce complications and costs of the treatment of perineal lesions [16–18].

PEMFs technology has been reported to accelerate the healing of cutaneous wounds and ulcers by modulating the course of processes such as inflammation, tissue repair, and establishment of homeostasis [18, 35–38]. Our study confirmed that PEMS technology could speed up the complete healing of postpartum perineal lacerations and episiotomies.

Postpartum perineal tear or episiotomy can be complicated by wound dehiscence in around 0,1%-0,2% of cases. This complication is among the main reasons driving women towards an elective cesarean section for maternal request [51, 52]. The possible therapeutic options are currently medical conservative approach or an early wound re-suturing [53–56]. This pilot study provides evidence that this technology could be tested in these severe complications frequently associated with permanent dysfunctions of the pelvic floor.

This study had several strengths: the study population presented a variety of obstetric perineal traumas, spanning from mediolateral episiotomies to different degrees of spontaneous lacerations, either occurring alone or associated with episiotomies, thus providing a wide range of lesions to be observed during healing process. Although the intervention and the control group were not the result of randomization, the number of complicated lacerations, grade 2 and grade 3 lacerations and complicated episiotomies was not significantly different between the two groups.

In order to evaluate the process of healing and, later, the sexual function, we used validated and established scales, like the REEDA scale and the FSFI questionnaire.

The main limitation of our study was the relatively small sample size which does not represent a wide population. In addition, the data derived by the FSFI questionnaire should be considered with caution given the number of dropouts and the close follow-up.

## Conclusion

The main finding of this study is that a biophysical therapeutic approach based on PEMFs technology has allowed a faster and complete healing of postpartum perineal lacerations and episiotomies. Our results highlight that this new technology may be effective also to ameliorate the sexual discomfort following postpartum perineal lesions, and we advocate to validate this innovative therapeutic approach in a multicenter prospective trial.

Overall, these data claim individualized clinical care on postpartum sexual recovery and long-term studies are needed to explore this crucial, and too often undermined, aspect of postpartum perineal well-being. Standardized follow-up protocols must be set in place to allow a better comparison with alternative therapeutic strategies and to allow the best choice both from a clinical and cost related point of view.
